# Impact of co-packaging oral rehydration salts and zinc on diarrhoea treatment dispensing behaviour in selected rural health facilities in Zambia

**DOI:** 10.1371/journal.pgph.0004342

**Published:** 2025-03-26

**Authors:** Simon Berry, Dario Domingo, Akufuna Ngenda, Jane Berry, Rohit Ramchandani

**Affiliations:** 1 ColaLife (charity number 1142516), London, United Kingdom; 2 Department of Mathematical Sciences, Durham University, Durham, United Kingdom; 3 UCD School of Mathematics and Statistics, University College Dublin, Dublin, Ireland; 4 Keepers Zambia Foundation, Lusaka, Zambia; 5 Department of International Health, Johns Hopkins Bloomberg School of Public Health, Baltimore, Maryland, United States of America; 6 United Nations University - Institute for Water, Environment and Health, Richmond Hill, Canada; 7 Balsillie School of International Affairs, Waterloo, Canada; PLOS: Public Library of Science, UNITED STATES OF AMERICA

## Abstract

Co-packaged oral rehydration salts (ORS) and zinc for the treatment of childhood diarrhoea was added to the World Health Organization’s Essential Medicines List in 2019, to help address the persistently high mortality and morbidity associated with diarrhoea in children under 5 years of age and the low uptake of the recommended co-therapy. However, little empirical evidence exists on how co-packaging impacts dispensing practices in low-resource settings. Here, we present findings from a study conducted in Mongu District, Zambia, aimed at evaluating the effect of introducing a co-pack containing ORS and zinc on dispensing behaviour at rural health facilities. Data from dispensing records were collected before and after the introduction of the co-pack, in 2016 and 2017, respectively, from seven government health facilities. We used multilevel logistic regression to account for the fact that the data is clustered by health facility and to address potential intraclass correlations in dispensing practices within the same facility. The results indicate an overall odds ratio of 8.42 (95% CI: 5.47-12.9) for the dispensing of both ORS and zinc together, for 2017 versus 2016, along with a significant reduction in the variability of dispensing practices between facilities (once the co-pack factor was included). Additionally, the data suggest that less well-resourced facilities experienced the greatest benefit from the introduction of the co-pack. These findings provide valuable insights into the potential of co-packaging to foster appropriate diarrhoea-treatment dispensing practices in resource-limited settings. As such, they provide a foundation for further research to validate them on geographical scales beyond the district level.

## 1. Introduction

Two decades ago, in 2004, the World Health Organization (WHO) and UNICEF first called for childhood diarrhoea to be treated with both oral rehydration salts (ORS) and zinc sulphate [1], estimating, at that time, that 1.5 million children under five years of age were dying of diarrhoea annually. Co-therapy with zinc sulphate was recommended following studies that found that its administration reduced the severity and duration of diarrhoea in addition to offering a protective effect from reinfection [2,3]. ORS has been recommended to prevent death through rehydration since the 1970s [4]. While mortality rates due to diarrhoea have improved over the past 20 years, coverage of diarrhoea cases with both ORS and zinc still stands at just 18% in sub-Saharan Africa and 19% globally [5] despite some efforts to improve it, particularly through supply chain stimulation coupled with awareness raising and policy guidance [6–17]. Diarrhoea continues to cause an estimated 444,000 deaths annually, equating to 9% of all deaths among children under the age of 5 years worldwide [5].

In 2019, WHO accepted an application from key actors [18] and amended its Model List of Essential Medicines to include, for the first time, co-packaged ORS and zinc (ORSZ) for the treatment of diarrhoea [19]. Key points in the application included the potential of co-packaging to increase coverage and improve adherence to the combined therapy as, particularly in low-resource settings, sachets of ORS and zinc sulphate tablets may not be available at the same time and in the right ratios, or health personnel may not know of the recommendation to dispense both medicines together. Co-packaging was accepted by WHO as a valid approach to increase coverage of the co-therapy and is gaining ground as a possible intervention, adaptable to local contexts, to address this implementation gap [8,12]. UNICEF states that over 20 countries have introduced co-packaged ORSZ in the public sector and now advocates that countries use only co-packaged ORS and zinc to promote adherence and optimal treatment [12]. However, only a few studies have been conducted to explore or validate the effect of co-packaging ORS and zinc on the coverage of ORSZ combined therapy [9–11,13–16,20]. Black (2019) concluded that co-packaging can rapidly equalise the use of ORS and zinc [8], and some governments are reportedly moving towards a preference to procure co-packs [9–11,14]. Few studies, however, have specifically explored the effect of co-packaging on dispensing practices.

Here, we investigate whether the introduction of a co-pack containing ORSZ at remote rural government health facilities in Zambia affected the dispensing practices of health personnel. We compare dispensing practices at various health facilities before and after the introduction of a new co-pack by the Zambian government. All facilities were supported by the District Health Office in Mongu town (Mongu District). The intervention formed part of a larger trial and subsequent scale-up initiatives of co-packaged ORSZ, which were conducted in Zambia from 2011 to 2018 and are reported elsewhere [14–16,21]. The aim of our analysis was to assess whether the introduction of the co-pack significantly increased the likelihood of a child under five years of age, who is affected by diarrhoea, being prescribed and dispensed both ORS and zinc, on presenting with diarrhoea at a facility.

## 2. Methods

### 2.1. Context

Mongu District is a remote, rural district in Zambia’s Western Province. In 2022 it had a population of 198,000, a 3.7% increase since 2010. The district town lies at the end of the 590 km Great West Road from Zambia’s capital, Lusaka.

A convenience sample of health facilities supported by the District Health Office in Mongu were selected, with all but one of the facilities falling within Mongu district (Mweeke facility, although supported by the Mongu District Office, technically sits just over the border, in the neighbouring district of Limulunga). In Mongu our researchers were able to benefit from well-equipped local staff employed by a partner non-governmental organisation, who had already been trained within the wider project. Moreover, the researchers had access to data relating to the recorded stock position for ORS, zinc and co-packaged ORSZ across 29 of the approximately 40 health facilities in or supported from Mongu at that time.

The health service delivery system in Zambia has three levels. The first level comprises community-level facilities, including urban and rural health centres (UHCs and RHCs) and health posts (HPs). The second level comprises provincial and general hospitals, and the third level comprises central and specialist hospitals focused on tertiary care. The focus of this study was UHCs, RHCs and HPs, which comprise the frontline facilities for primary healthcare. Of the 29 facilities initially surveyed to assess their ORS and zinc stock status, 3 were UHCs, 18 were RHCs and 8 were HPs.

Each type of facility in Zambia has differing resources and staffing levels. Most facilities are typically in a poor state of repair and under-resourced [22]. While RHCs should be staffed by one each of a midwife, nurse, environmental health officer, clinical officer and public health nurse, in practice this is often not the case. UHCs are typically larger and better resourced than RHCs; in addition to the staff recommended for RHCs, they should have a medical doctor, pharmacist, laboratory personnel, physiotherapist and nutrition adviser. HPs typically comprise a single-room building. While they should have access to similar allied health staff as RHCs, they are often serviced by minimal staff, for example one community health assistant who typically has received just one year’s training. A community health assistant may spend 80% of their time in the community and just 20% at a HP [23]. In the absence of a community health assistant, an untrained volunteer or an environmental health officer may see patients and dispense treatments.

All the centres that participated in this study were supplied with stock through the government’s usual distribution system via Medical Stores Limited (now known as Zambia Medicines and Medical Supplies Agency, ZAMMSA), working in collaboration with the local district council. All medicines were dispensed free of charge from government health facilities. In Zambia, treatment seeking behaviour is strongly biased towards the public sector. The most recent Demographic and Health Survey (DHS 2018) [24] found that 93% of children with diarrhoea were taken to public sector health facilities for advice or treatment, while only 5.6% were taken to private sector facilities. Less than 1% of advice or treatment for diarrhoea is sought from private shops or markets. The most common source of advice or treatment, for those who seek it, is a government health centre (67%), followed by a government health post (18%).

### 2.2. Data collection

Our objective was to compare dispensing practices among government health facilities on the frontline of primary healthcare, before co-packaged ORSZ was introduced, in 2016, and after it became available, in 2017. We selected the month of October to make this comparison as it is the peak month for diarrhoea cases [25]. In undertaking this study, we made no attempt to influence the normal stocking procedures carried out by Zambia’s Ministry of Health [14]. Instead, in November 2017 we surveyed 29 primary health facilities to retrospectively assess their stock status during October 2016 and October 2017. As a result of this process, we identified five facilities with inadequate stock records, and these were excluded from the analysis. Among the remaining facilities, a fully stocked facility was defined as being one satisfying the following two criteria: i) having ORS and zinc (packaged separately) throughout the month of October in 2016, and ii) having ORS and zinc (packaged separately) as well as co-packaged ORSZ throughout the month of October in 2017. A fully stocked facility had stock at the beginning and end of the month and experienced no stock-outs during the month. Seven of the 24 facilities met these stocking criteria, and these were the source of the dispensing data used in this study. These seven facilities comprised one UHC, three RHCs and three HPs. All data were collected by a single enumerator between 23 November 2017 and 28 December 2017.

The enumerator used the existing Ministry of Health dispensing ledger to establish actual dispensing behaviour and recorded this on a paper-based tally sheet. A bespoke, tablet-based form, developed using the CommCare platform [26], was used to collect basic data (*e.g.*, the name of the facility and hosting member of staff) and the tablet’s GPS capability automatically recorded the facility’s location. At the end of the process a picture was taken of the two paper-based tally sheets, one each for 2016 and 2017, and these were uploaded to the CommCare server as attachments to the CommCare form for later review and validation.

### 2.3. Inclusivity in global research

Additional information regarding the ethical, cultural, and scientific considerations specific to inclusivity in global research is included in the Supporting Information (S1 Checklist).

### 2.4. Statistical methods

For the purposes of this study and based on the global WHO/UNICEF recommendation, we considered the dispensing of diarrhoea treatment as correct when both ORS and zinc were provided to the patient or their caregiver, either packaged individually (the only option in 2016) or as a co-pack. Accordingly, we clustered dispensing behaviour into a dichotomous response, distinguishing between ‘correctly-dispensed’ and ‘incorrectly-dispensed’ cases. The aim of the present work was to investigate whether the correct dispensing rate (CDR) underwent a change, following the introduction of the co-pack.

The data presented a two-level structure, with individual dispensing behaviour nested within facilities. We analysed the data using multilevel logistic modelling, to account for potential correlation in dispensing practices within the same facility [27] and to draw reliable inference from health facilities with fewer cases. Specifically, we used two models, to achieve two different goals: a simple random-effect model to estimate the CDR of each facility in either of the two years; a more complex mixed-effect model to quantify the impact of the co-pack introduction on the facilities’ CDR and odds ratio. Details on the models and the rationale behind the choices follow. All models were fitted in R, version 4.3.2, via the lme4 package.

All models trained in this study were logistic regression models with dichotomous response identifying correct/incorrect dispensing behaviour. Thus, each model predicted the odds of correct dispensing (the ratio between the probability of correct dispensing and the probability of incorrect dispensing) as a function of relevant predictors. The second of the following two formulas (one the inverse of the other) then allowed to expressed the odds of correct dispensing as CDR:


$$\text{Odds}=\frac{CDR}{1-CDR}\text{and}CDR=\frac{\text{Odds}}{1+Odds}.$$ (1)

In the random-effect logistic model, facilities were included as random effect and no fixed effect predictors were included. We fitted the model, separately, on each of the two year’s data. This enabled us to estimate the odds (and thus the CDR) of the single facilities, and the associated uncertainty, in each of the two years separately (2016, no co-pack available; 2017, co-pack available). We notice that, through the random-intercept approach, each facility’s CDR was inferred by adjusting the overall CDR to the data observed in that facility, thus compensating for the limited amount of data of some individual facilities.

After estimating the CDR of the single facilities, we fitted a mixed-effect logistic regression to the whole data set. The model included a factor for co-pack availability as fixed effect and one for facilities as random effect. In other words, a linear predictor was used to model the log odds of correct dispensing, with fixed slope quantifying the effect of co-pack availability, and random intercept varying among facilities. The choice allowed us to explicitly model the effect of the co-pack introduction on dispensing practices, while accounting for random variation between facilities due to factors which could not be measured.

For both models, we quantified the average degree of correlation among dispensing practices within the same facility via the intraclass correlation coefficient (ICC). We computed the ICC using the simulation approach described in [28], as appropriate to the case of binary-outcome logistic models. A near-zero ICC indicates that a lack of within-facility correlation may be assumed for modelling purposes, and thus that a classical logistic model (with no random effects) may also be used. We will consider this option during the exposition of the results, according to the outcome of the multi-level model.

### 2.5. Ethics approval

Overall clearance for data collection for this study fell under the KYTS Programme of which it formed a part. Implementation of the project in Zambia was authorised by national and district government health authorities and through local chiefs. Ethics approval was obtained from ERES Converge in Zambia (IRB No. 00005948). No personal data were collected as part of this study. We did not engage with human participants; we only accessed archives of dispensing records and did not use or access any information that could identify individual participants during or after data collection.

## 3. Results

For each of the seven facilities that met our inclusion criteria, Table 1 reports the number of cases for which a diarrhoea treatment was dispensed, and the proportion of these that were correctly dispensed (*i.e.*, that included both ORS and zinc). The data is shown for each of the two years separately (2016 and 2017, before and after the co-pack introduction, respectively), with the last row summarising results across all facilities.

**Table 1:** Observed diarrhoea cases and correctly dispensed cases (in absolute and relative terms) in October 2016 and October 2017, by facility and overall.

	2016 (before co-pack)			2017 (after co-pack)				
	All Cases	Correctly dispensed	CDR	All Cases	Correctly Dispensed	CDR	Co-packs	% of co-packs out of correctly dispensed
Mulambwa UHC	67	46	69%	230	189	82%	167	88%
Lukalanya RHC	12	3	25%	30	29	97%	17	59%
Lukweta RHC	23	3	13%	22	22	100%	22	100%
Nalwei RHC	18	12	67%	42	35	83%	24	69%
Kaande HP	14	2	14%	14	12	86%	12	100%
Mweeke HP	10	2	20%	26	26	100%	26	100%
Nasange HP	27	7	26%	30	29	97%	29	100%
**Total**	**171**	**75**	**44%**	**394**	**342**	**87%**	**297**	**87%**

For 2017, the number of dispensed co-packs and their percentage relative to all correctly dispensed cases are also shown.

UHC, urban health centre; RHC, rural health centre; HP, health post.

CDR, correct dispensing rate.

**Fig 1:**
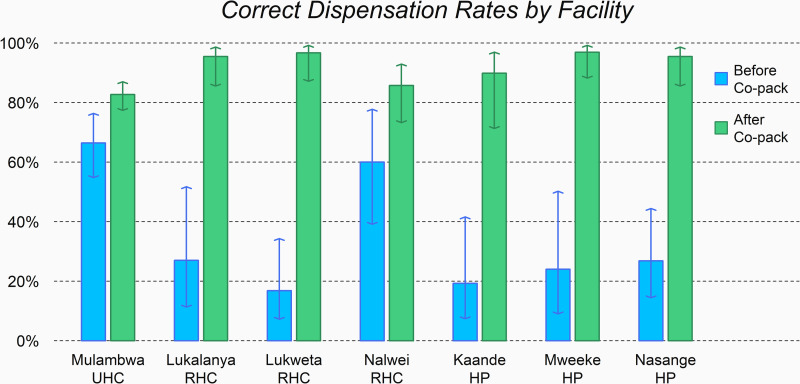
Comparison of the CDR at each of the seven health facilities, before and after the introduction of co-packaged ORSZ. The CDRs (bars) and associated CIs (whiskers) are estimated through random-intercept models with correct dispening as outcome and facilities as random intercept (Table 2).

The overall CDR in the sample, as computed across all facilities, increased from 44% to 87% following the introduction of the co-pack. As Table 1 shows, the CDRs of individual facilities vary around the aforementioned percentages. We estimated the underlying CDR of each facility, separately in each year, via a random-intercept model with correct dispensing as binary outcome and facilities as random intercept. Table 2 presents the results of fitting the model to the 2016 data (before co-pack availability) and to the 2017 data (after co-pack availability). Fig 1 offers a graphical illustration of the change in the estimated CDRs (bars) and associated CIs (whiskers), between the two years.

**Table 2:** Results of the random-intercept model with correct dispensing as binary outcome and facilities as random intercept.

Model	Intercept Estimate	ICC	Number of observations
2016 model (before co-pack)	−0.761	0.161	171
2017 model (after co-pack)	2.714	0.077	394

The model is fitted separately on each year’s data. The intercept estimate represents the estimated common log odds of correct dispensing, across all facilities, in the relevant year.

The bar plot in Fig 1 reveals that all facilities increased their CDR following the introduction of the co-pack. The varying amplitude of the CIs reflects different sample sizes at each facility in each year (narrower CIs generally correspond to larger sample sizes). Notice that, with the exception of Nalwei RHC, the CIs for the two years at any facility do not overlap with each other, indicating that the increase is significant at least at the 5% level. At Nalwei RHC, a small overlap can be detected. Additionally, we note that for Mulambwa UHC and Nalwei RHC the increase is present, but more contained than for the remaining five facilities. In contrast to the other facilities, these two had relatively high CDRs prior to the co-pack introduction.

As indicated by Table 2, the simulated ICC for the models is equal to 0.161 in 2016 and 0.077 in 2017. This reveals that around 16% and 8% of the total variability in dispensing practices in the two years can be attributed to differences between the facilities, while most of the variability comes from varying dispensing practices within facilities.

To quantify the change in CDR that took place following the introduction of the co-pack, and its statistical significance, we fitted a mixed-effect model to our data. The model has co-pack availability as fixed effect and facilities as random intercepts.

Table 3 summarises the results of the fit. The common odds ratio across facilities is estimated to be 8.42 (95% CI: 5.47 - 12.9). The results suggest that the odds of correct dispensing at least quintupled following the introduction of the co-pack. The odds ratio estimate is highly significant, as is the whole model: a likelihood ratio test of the model versus an intercept-only model (both with fixed intercept, and with facility-dependent random intercept) returns a p-value<0.0001.

**Table 3:** Mixed-effect model results (co-pack availability as fixed effect, facilities as random intercepts).

	Coefficient	St. Error	p-value	Odds Ratio	95% CI
Co-pack	2.130	0.214	<0.0001	8.42	5.47–12.9
Intercept	−0.247	0.154	0.109	-	-

Likelihood ratio test vs intercept-only model: p<0.0001.

It is interesting to note that we estimate a near-zero ICC ($\sim 10^{-5}$) for the model. This reveals that, once a factor for co-pack availability is taken into account, the data behaves as though dispensed cases within facilities were independent. This would make a standard logistic regression, with co-pack availability and facility as covariates, also appropriate to the dataset. In additional investigations we do not detail here, we have also explored this option: as expected, the results confirm the outcomes of the above mixed-effect model, hardly adding any relevant information to it (highly significant co-pack factor, not significant facility in the presence of co-pack).

From Fig 1, we note, however, that two groups of facilities may be identified, according to the CDR that these displayed prior to the co-pack introduction: Mulambwa UHC and Nalwei RHC on the one hand (high CDR prior to the introduction of the co-pack, around 60%) and the remaining five facilities on the other (low CDR prior to the introduction of the co-pack, below 30%). We thus decided to fit a classical logistic regression model to the data of each of the two groups separately. The model included co-pack availability and facility as covariates. This aimed to investigate whether the estimated odds ratios in the two cases would be different from each other, while no significant difference would be detected between facilities in the same group.

The results confirm the above intuitions. The odds ratio is estimated to be 2.17 (CI: 1.24 - 3.77, p=0.006) for the high-baseline-rate group, and 128 (CI: 44 - 493, p<0.0001) for the low-baseline-rate group. Note the much wider CI in the second case, due to the lower amount of data and a much greater central estimate of the odds ratio. While the odds ratio estimates are very different for the two groups, no significant difference shows up between facilities of the same group. The p-value for the difference in log odds between Mulambwa UHC and Nalwei RHC is 0.96. Similarly, all p-values for the difference in log odds between pairs of facilities of the second group are higher than 0.19. We speculate on the possible causes of this in the discussion.

## 4. Discussion

The transition from individual ORS and zinc products to a co-packaged format, particularly within the public sector, has emerged as a pivotal strategic initiative across several countries, significantly elevating combined ORS and zinc coverage levels [8–11,13,14]. This is one of the first studies to look specifically at the influence of co-pack introduction on dispensing practices at the facility level.

Overall, across the seven frontline health facilities analysed in this study, the introduction of co-packaged ORSZ increased the rate of dispensing of the recommended treatment for diarrhoea (ORS together with zinc) from 44% to 87%, corresponding to an 8.42 increase in odds ratio (95% CI: 5.47 - 12.9, p<0.0001). Disaggregation of the overall improvement showed that all facilities in the study improved their dispensing of the recommended treatment for diarrhoea following the introduction of co-packaged ORSZ. The largest increases in correct dispensing rate (CDR) were seen in the less well-resourced HPs (Kaande, Mweeke, Nasange), as well as in two of the three RHCs (Lukalanya, Lukweta). Despite a lower number of cases across the five facilities (86 in 2016, 122 in 2017), the estimated common odds ratio for the facilities was highly significant (128, CI: 44 - 493, p<0.0001). The increase in odds ratio was present and statistically significant also for the other two facilities (Mulambwa UHC and Nalwei RHC), although more contained (2.17, CI: 1.24 - 3.77, p=0.006). This suggests that co-packaging ORSZ may be particularly helpful in less well-resourced settings, where fewer and/or less-trained staff may be available.

The retrospective nature of this study meant that qualitative interviews with staff were not possible. Key informant interviews would have been helpful to understand certain aspects of the dispensing behaviour observed at Mulambwa UHC and Nalwei RHC. These two facilities displayed the highest CDRs before the introduction of the co-packs and benefited the least from their introduction. They also dispensed, in 2017, a relatively low percentage of co-packs out of all correct treatments: 88% and 69% respectively, compared to the 100% at four of the other five facilities (Table 1). Various factors could explain this. It may have been that the staff at these facilities were better trained [29,30] and that dispensing was determined more closely by expiry dates such that older stocks of ORS and Zinc, packed separately, were dispensed prior to the newly arrived co-packs. It may have been that more experienced or better trained staff had less need to embrace the convenience of the co-pack. However, our data does not include the qualitative information required to draw robust inference on potential reasons behind this observation.

We note that, while improved dispensing can be expected to translate into improved coverage, other factors such as user preferences [31], rational use, design and adherence [15] are known to influence effective coverage [8,32,33]. Nevertheless, the CDR of 87% seen in this study on a sample of about 400 cases compares very favourably to the Zambian national average coverage of ORS and zinc (34%) [24].

We conclude by acknowledging some limitations of the present study. Mongu District was not selected to be representative of all rural districts in Zambia, the choice was mainly dictated by contingent situations pertaining to the wider project discussed at the end of the Introduction section. The restricted geographical focus limits the extension of the validity of the described results to larger, national or international, geographical scales. Furthermore, the retrospective design of the study precluded sample size estimation prior to data collection, limiting our ability to correct for imbalances between facilities and ensure adequate power to detect predefined effect sizes. Despite these limitations, the results presented here offer robust insights into the research question, achieving high statistical significance with the available data. We believe the evidence is sufficiently promising to warrant further research to externally validate these findings in different contexts and at broader geographical scales. Future studies could also incorporate interviews with dispensing staff to better understand additional factors influencing dispensing behaviour.

## 5. Conclusions

Our analyses show that, across the examined facilities, the introduction of co-packaged ORS and zinc was highly effective in increasing the number of cases where the globally recommended treatment for diarrhoea was dispensed. The most significant improvements were observed in less well-resourced settings. We encourage continued research to assess the impacts of co-packaging and to further validate these findings on a broader scale.

## Supporting information

S1 Checklist Inclusivity in global research. (PDF)
